# Notch Signaling in B Cell Immune Responses

**DOI:** 10.3389/fimmu.2020.609324

**Published:** 2021-02-05

**Authors:** Matthew Garis, Lee Ann Garrett-Sinha

**Affiliations:** Department of Biochemistry, State University of New York at Buffalo, Buffalo, NY, United States

**Keywords:** B cell, notch, jagged, delta-like ligand, differentiation

## Abstract

The Notch signaling pathway is highly evolutionarily conserved, dictating cell fate decisions and influencing the survival and growth of progenitor cells that give rise to the cells of the immune system. The roles of Notch signaling in hematopoietic stem cell maintenance and in specification of T lineage cells have been well-described. Notch signaling also plays important roles in B cells. In particular, it is required for specification of marginal zone type B cells, but Notch signaling is also important in other stages of B cell development and activation. This review will focus on established and new roles of Notch signaling during B lymphocyte lineage commitment and describe the function of Notch within mature B cells involved in immune responses.

## The Canonical Notch Signaling Pathway

Notch signaling is initiated by the interaction of cell-surface-bound Notch ligands (members of the Jagged and Delta-like families of proteins) to Notch receptors on adjacent cells. The Notch family of receptors and their ligands are highly evolutionarily conserved proteins, found in all metazoan animals tested (1). In mammals, there are 4 Notch receptors (Notch1, Notch2, Notch3, and Notch4) and 5 Notch ligands (Jagged1 (Jg1), Jagged2 (Jg2), Delta-like ligand1 (Dll1), Delta-like ligand 3 (Dll3) and Delta-like ligand 4 (Dll4) (2). The Notch pathways are involved in developmental decisions and cell fate choices in a wide variety of tissues in mammals and other organisms.

**Figure 1:**
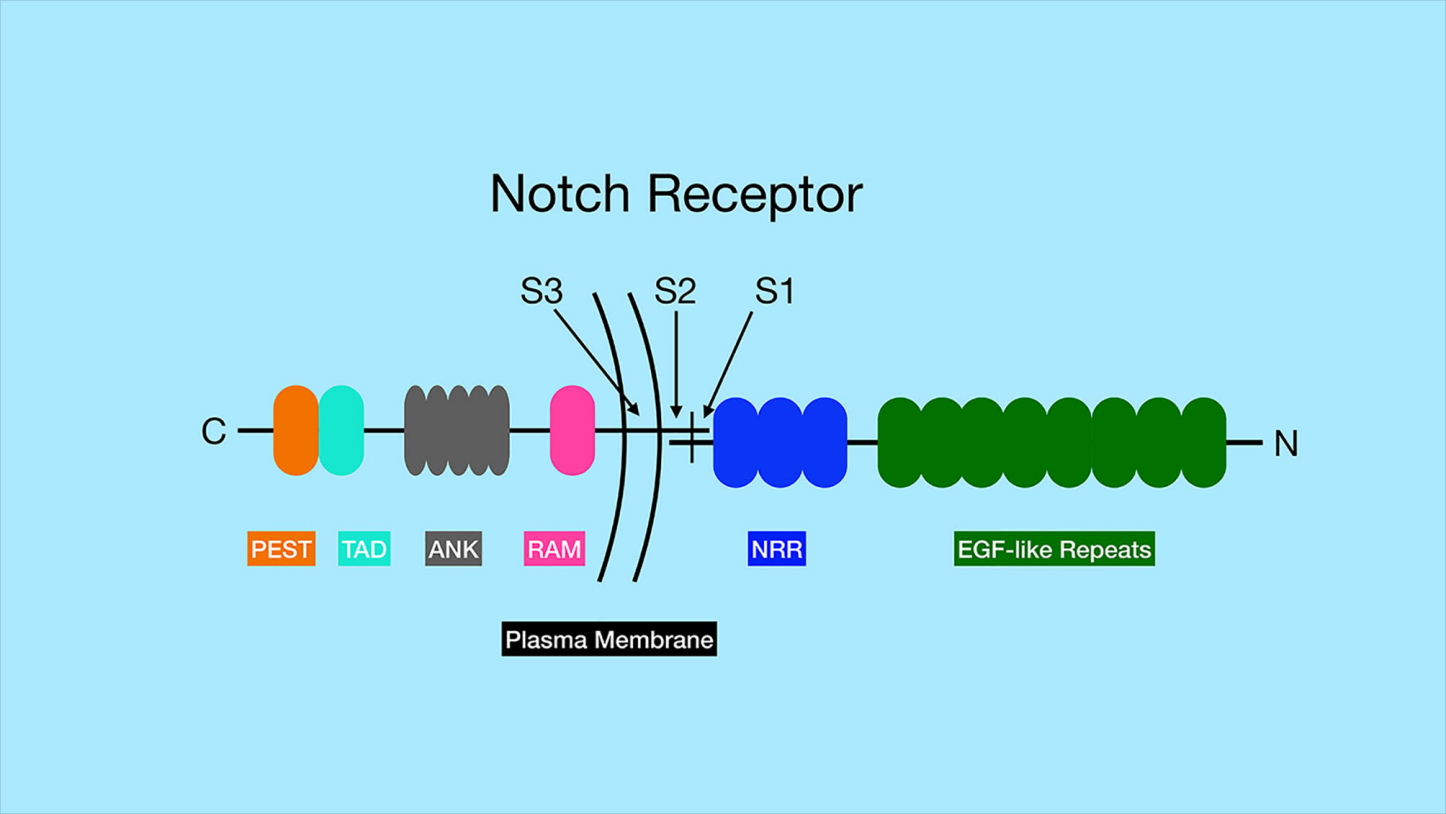
General structure of Notch family of receptors. Notch receptors are proteolytically processed into two separate sub-parts that remain non-covalently associated. The N-terminus of the protein, located outside the cell, contains a series of EGF-like repeats (dark green) involved in ligand binding. The numbers of EGF-like repeats differ between different Notch family members. The extracellular portion of the Notch receptors also contains the negative regulatory region (NRR, blue), which prevents Notch signaling until ligand binds. The site of ADAM protease cleavage (Site 2 or S2) is located close to transmembrane domain. The transmembrane domain contains the site of *γ*-secretase cleavage (Site 3 or S3). The intracellular region of the Notch receptors contains an RBP-J associated molecular domain (RAM, pink), a series of ankyrin repeats (ANK, gray), a transactivation domain (TAD, aqua) and a proline-serine-threonine rich domain (PEST, orange). Note, that Notch3 and Notch4 lack the TAD domain, but contain the other domains indicated in the figure.

**Figure 2:**
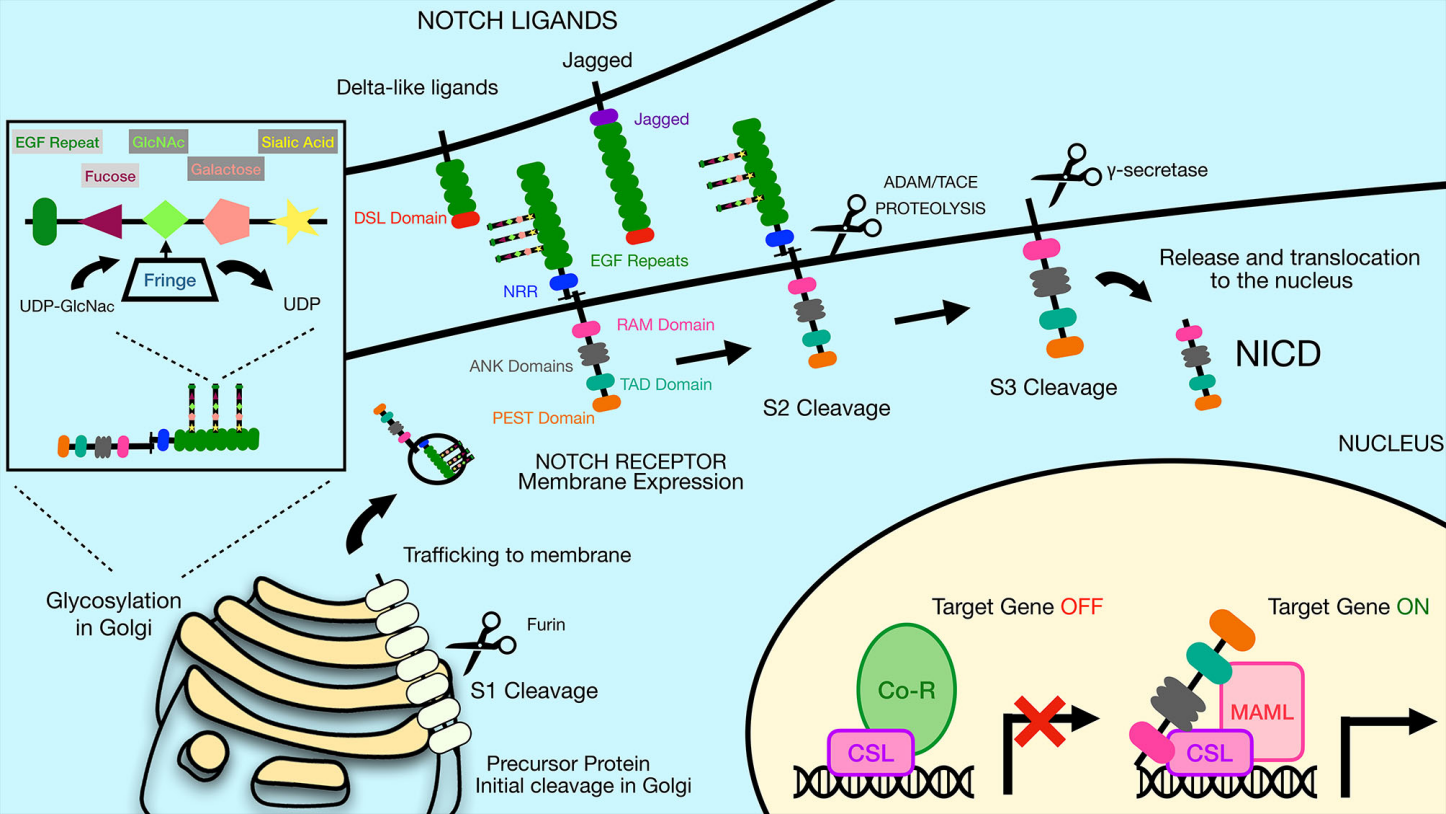
The Notch signaling pathway is mediated by a series of proteolytic events. Notch receptors are generated by ribosomes bound to the endoplasmic reticulum (ER) and trafficked through the Golgi to the plasma membrane. During the time in the Golgi, Notch receptors are cleaved at Site 1 (S1) by furin-like proteases to generate two sub-parts that remain non-covalently associated. The N-terminal subunit can be O-glycosylated *via* the activity of a series of glycosyltransferase enzymes, including members of the Fringe family that catalyze addition of N-acetylglucosamine residues residues to the glycan chain. Once at the plasma membrane, Notch receptors are inactive unless bound by ligand on adjacent cells. Notch ligands constitute two families, the Delta-like ligands and the Jagged family ligands. Both types of ligands contain a conserved Delta-Serrate ligand (DSL) domain that mediates binding to Notch receptors. Upon ligand binding, Notch receptors undergo cleavage by ADAM family proteins at Site 2 (S2). This allows subsequent cleavage by *γ*-secretase at Site 3 (S3), releasing the Notch intracellular domain (NICD). The liberated NICD is translocated into the nucleus to bind to RBP-Jκ along with the coactivator Maml, leading to activation of target gene expression. Prior to binding of NICD and Maml, RBP-Jκ is associated with co-repressor proteins that prevent transcription of target genes. DSL, Delta/Serrate/Lag2; UDP, Uridine diphosphate; GlcNAc, N-acetylglucosamine; NICD, Notch intracellular domain; Maml, mastermind-like protein; Co-R, corepressor protein complexes.

The Notch receptors are synthesized as precursor proteins and are first cleaved in the Golgi at a site referred to as Site 1 (**S1**), resulting in two fragments of the protein that non-covalently associate with one another (**Figure 1**) (3). The N-terminal portion contains the majority of the extracellular region of the protein, while the C-terminal portion contains a small region of the extracellular domain, the transmembrane and intracellular domains of the protein. The extracellular domain of Notch receptors contain numerous EGF repeats that function in ligand binding. NMR studies have shown the extracellular region of Notch receptors to be an elongated structure that sticks out into the extracellular space awaiting ligand binding at EGF domains in a calcium-dependent manner (4, 5). The EGF repeats are followed by a negative regulatory region (NRR) which prevents premature ligand-independent activation of Notch receptors by occluding a proteolytic cleavage site. The NRR domain also mediates the non-covalent interaction of the two fragments of the Notch receptor generated by cleavage at S1 (6). The intracellular region of Notch receptors is comprised of a RBP-Jκ association module (RAM) domain, seven ankyrin (ANK) repeats flanked by two nuclear localization signals, a transactivation domain (TAD), and a proline/glutamic acid/serine/threonine-rich motifs (PEST) domain that promotes degradation (**Figure 1**). Once Notch receptor and Notch ligand bind, a force is generated which is thought to unfold the NRR domain and allow cleavage at a proteolytic site referred to as Site 2 (**S2**) by ADAM (a disintegrin and metalloproteinase domain) family metalloproteases (7, 8) (**Figure 2**). The pulling force is mediated by internalization of the Notch ligand by endocytosis while interacting with Notch receptors on adjacent cells. The endocytosis process internalizes Notch ligands while tugging on the Notch receptor and unraveling the NRR domain. Measurements of the force necessary to unravel Notch receptors showed that it takes roughly 4 to 9pN to activate the receptor (9, 10).

**Figure 3:**
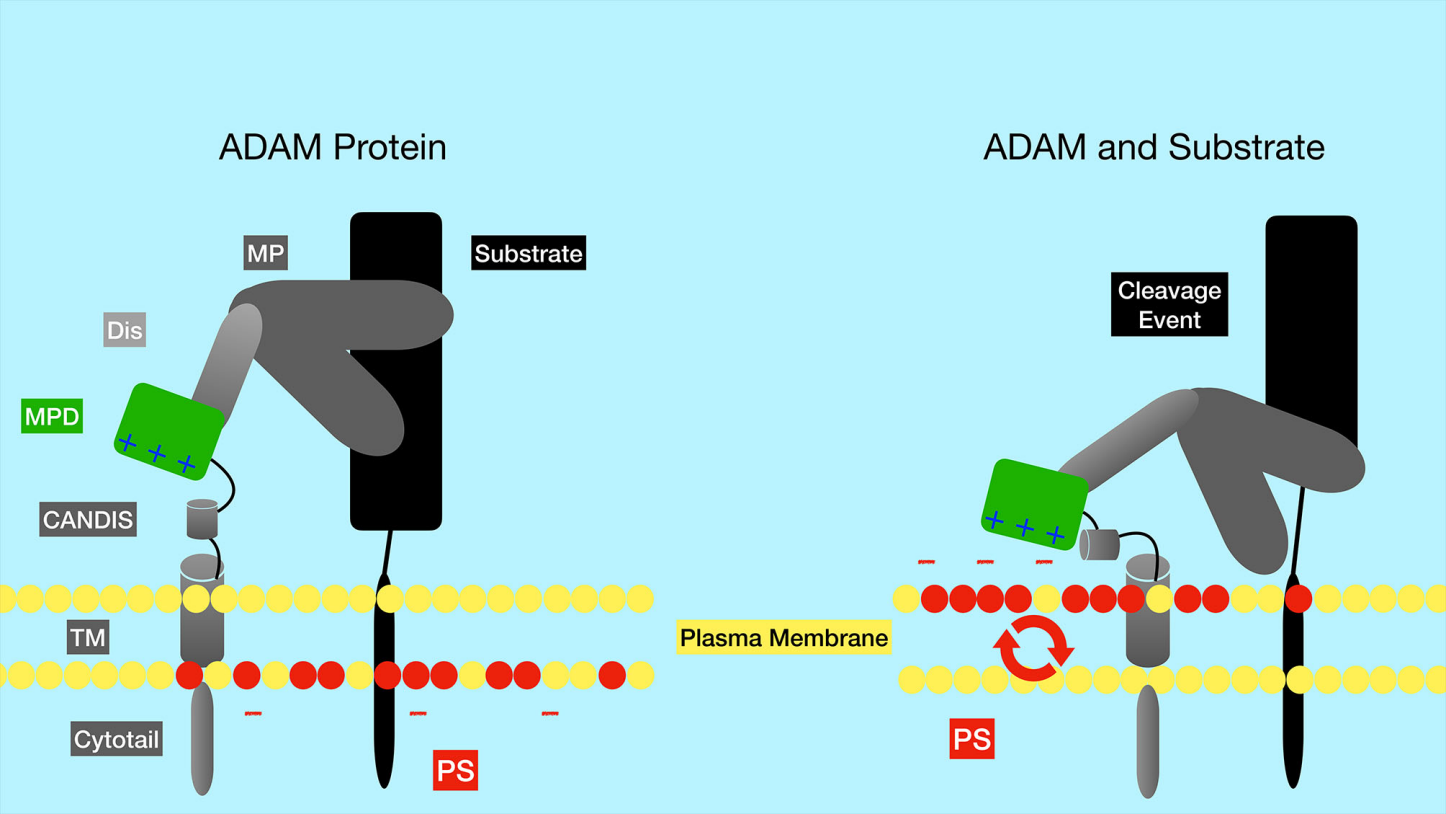
Regulation of the ADAM metalloproteinases by phosphatidyl serine of the plasma membrane. The negatively charged membrane phospholipid phosphatidyl serine (red circles) is typically found in the inner leaflet of the membrane, but can be flipped to the outer leaflet by the action of phospholipid scramblase enzymes such as ANO6. Positively charged amino acid residues in the ADAM10 stalk domain (CANDIS) interact with negatively charged phosphatidyl serine, altering the conformation of ADAM10. These alterations are thought to bring the catalytic center of the enzyme closer to the plasma membrane and to move an inhibitory loop out of the catalytic site, thereby activating ADAM10 protease activity. PS, phosphatidyl serine; TM, transmembrane domain; CANDIS, Conserved ADAM Dynamic Interaction Sequence; MPD, membrane proximal domain; Dis, disintegrin domain; MP, metalloproteinase domain.

The ADAM family contains metalloproteases that function as sheddases as they cleave and “shed” extracellular portions of transmembrane proteins. While the ADAM family has approximately 30 identified members in mice and humans, ADAM10 and ADAM17 have been shown to be particularly important in Notch activation. ADAM17 was the first family member shown to be able to cleave Notch1 at the S2 site (7). However, ADAM17 knockout mice lack embryonic defects associated with impaired Notch signaling. Later studies in mouse embryonic fibroblasts (MEFs) showed that ADAM10 was the predominant ADAM family member that cleaves Notch in response to ligand binding (11). Furthermore, ADAM10 knockout mice have defects in Notch signaling pathways, consistent with an important role for ADAM10 in Notch activation (8, 12, 13). In fact, while many proteases can cleave Notch in a ligand-independent manner, ADAM10 is required for Notch1 cleavage in a ligand-dependent manner, while both ADAM10 and ADAM17 can cleave Notch1 in a ligand-independent manner (14). Exposure of the negatively charged phospholipid phosphatidylserine (PS) on the outer leaflet of the membrane is required to induce ADAM10 activity (**Figure 3**) (15). The ability of externalized PS to induce ADAM10 activation depends on interaction of positively charged amino acid residues in the ADAM10 stalk domain (the CANDIS domain), which interact with negatively charged phosphatidyl serine headgroups. These interactions are thought to bring the catalytic center of the ADAM10 close to the plasma membrane and to move an inhibitory loop out of the catalytic site, thereby activating ADAM10 protease activity (15). Patients with the bleeding disorder Scott syndrome have a mutation in the calcium-dependent phospholipid scramblase Anoctamin-6 (ANO6, also called TMEM16F), which flips phosphatidyl serine from the inner to outer leaflet of the membrane. B cells from Scott syndrome patients lack ADAM10 sheddase activity due to a failure to expose PS (15). Interestingly, in normal B cells BCR ligation leads to transient PS exposure (16, 17), suggesting that B cell activation through antigen receptors may lead to an enhanced ability for Notch signaling. The BCR-dependent exposure of PS may rely on ANO6 activity, since treatment of B cells with a calcium ionophore resulted in ANO6-dependent PS exposure (18).

Following ADAM cleavage at the S2 site, a membrane-tethered intermediate known as Notch extracellular truncation (NEXT) is formed, which in turn is a substrate for the multi-subunit protease complex *γ*-secretase (2). *γ*-secretase cleaves Notch receptors at Site 3 (**S3**), which frees the intracellular domain of the Notch receptors (NICD) to allow them to translocate to the nucleus (**Figures 1** and **2**) (2). NICD can then interact with DNA binding protein RBP-Jκ (also called CSL or CBF1) *via* the RAM domain found in the NICD (2). The intracellular domains of Notch1 and Notch2 contain transcription activation domains that directly play a role in their ability to effect gene expression and cellular processes, while Notch3 and Notch4 lack similar transactivation domains (19).

Interaction of Notch receptors with Notch ligands can be modulated by O-linked glycosylation of the Notch receptors (2). These particular modifications are initiated by the enzyme POFUT1, which attaches fucose to specific serine/threonine residues in the EGF repeats of the extracellular portion of the Notch receptor. Additional sugar residues can be added to the fucose moiety by the action of glycosyltransferases, including members of the Fringe family proteins (**Figure 2**). In mammals, there are three Fringe enzymes referred to as Lunatic (Lfng), Manic (Mfng), and Radical Fringe (2). These Fringe proteins catalyze addition of N-acetylglucosamine residues to the glycan chain. Notch receptor glycosylation by Lfng and Mfng leads to enhanced activation by Delta-like ligands and reduced activation by Jagged ligands, while glycosylation by Radical Fringe enhances activation by all Notch ligands (20).

There is some evidence that different lymphoid cell types may differentially regulate Notch activity. For instance, lysates from human B cell lines and primary human B cells contain the NICD (p120 fragment) at levels similar to that found in T cell lysates, suggesting that Notch receptors are properly activated and cleaved in both cell types (21). But coimmunoprecipitation assays failed to find an association of NICD with RBP-Jκ in B cells, while this association was present in T cells. Interestingly, the EBNA2 protein of the EBV virus can associate with RBP-Jκ and result in transcriptional activation in the absence of NICD association (21). Although EBNA2 can compete with NICD for binding to RBP-Jκ, even B cells without EBV infection still failed to show an association of NICD with RBP-Jκ, suggesting that some aspect of the B cell intracellular environment prevents this association. As described in more detail below, Notch signaling also regulates various aspects of B cell maturation and function. Some of these processes have been shown to be dependent on RBP-Jκ, suggesting that the NICD-RBP-Jκ complex must form in B cells under certain conditions.

Mutations in the ANK repeats of Notch receptors abrogates Notch signaling (22). The ANK domains associate with cofactors such as Mastermind (Maml) forming a trimeric complex (RBP-Jκ, NICD and Maml) that is active for transcriptional stimulation (**Figure 2**). This complex was shown by crystal structure to bind directly to DNA (23). There are three mammalian Maml proteins, Maml1, Maml2 and Maml3. Both Maml1 and Maml2 are potent co-activators for all Notch family members, while Maml3 is a weaker activator and works most efficiently with Notch4 (24). Notch signaling induces expression of various target genes including those in the Hairy/Enhancer of Split (HES) family, such as Hes1, Hes5, Hey1, Hey2 and HeyL (25). These HES family proteins are basic helix-loop-helix proteins that repress the expression of other genes and thereby control differentiation processes in the cell. A summary of the major components of the canonical Notch signaling pathway described above are displayed in **Figure 2**. In addition to this canonical pathway of Notch signaling, Notch receptors can also transduce non-canonical signals as reviewed in Heitzler 2010 (26).

## Expression of Notch Receptors in B and T Cell Subsets

Notch receptors are expressed by both B cells and T cells in the spleen. Early studies using qPCR showed expression of Notch1 and Notch3 in mouse B cells at all stages tested, with the highest levels detected in pro- and pre-B cells in the bone marrow (27) (**Table 1**). However, this level of expression of Notch1 and Notch3 in B cells was 10-20x lower than the levels found in double negative thymocytes. The high expression of Notch1 and Notch3 in thymocytes is consistent with an important role development. Notch1 is required for specifying T cell fate (28), while Notch3 is is required for normal numbers of thymocytes (29). By qPCR, Notch2 is expressed at high levels in mouse B cells, particularly on mature B cell subsets in the spleen (27). Flow cytometry analysis with antibodies specific to Notch receptors showed that Notch1 and Notch2 proteins were both easily detectable in B220+ B cells from the mouse spleen (30). On the other hand, Notch3 was expressed at low levels in mouse B cells, while Notch4 was undetectable (**Table 1**). Further analysis showed that Notch2 was expressed at the highest levels in marginal zone B cells and marginal zone precursors (30), in keeping with its role in specifying this subset (see details below). Using a lacZ reporter knockin to the mouse *Notch2* locus, expression of the reporter gene was found to be low in transitional type I (T1) B cells in the spleen and in mature follicular B cells, but higher in follicular B cell precursors, transitional type II (T2) B cells and marginal zone B cell precursors and mature marginal zone B cells (31). Both BCR stimulation and stimulation with LPS have been shown to upregulate Notch1 expression in mouse B cells (32–34).

**Table 1:** Notch receptor expression in murine B cell subsets.

	Bone Marrow B cells		Splenic B cells			
	ProB	PreB	T1	T2	FoB	MZB
Notch1	+	+	+	+	+	+
Notch2	++	+	++	++	++	+++
Notch3	+	+	−	+	−	−
Notch4	−	−	−	−	−	−

(+) Weak expression, (++) moderate expression and (+++) high expression. (−) Little to no expression.

Notch receptor expression in human B cell subsets shows some differences from that seen in mice (**Table 2**). During development in the bone marrow, Notch1 is expressed at all stages tested, while Notch2 is preferentially expressed in late pre-B cells (35, 36). To our knowledge, expression of Notch3 and Notch4 in human bone marrow B cell subsets has not been examined. In human CD20+ B cells in peripheral blood and tonsil, both Notch1 and Notch2 are expressed, while expression of Notch3 is low and Notch4 is not expressed (37, 38). In tonsillar B cells, naïve and memory subsets express Notch1 and Notch2, but germinal center cells do not (39, 40). Expression on Notch receptors in human B cell subsets is summarized in **Table 2**.

**Table 2:** Notch receptor expression in human B cell subsets.

	Bone Marrow B cells					Peripheral B cells		
	Early B progenitor (CLP)	ProB	Early Pre-B	Late Pre-B	Immature B	Naïve B cells	Germinal Center B cells	Memory B cells
Notch1	+++	+++	+	++	+	++	+/−	++
Notch2	ND	−	−	++	−	++	+/−	ND
Notch3	ND	−	−	−	ND	−	−	ND
Notch4	ND	ND	ND	ND	ND	−	−	ND

(+) Weak expression, (++) moderate expression and (+++) high expression, (−) little to no expression, (+/−) expression seen in some studies and not in others.

Various cell types in the mouse spleen and lymph node have been reported to express Notch ligands (**Table 3**). Recently, Zhu et al. reported that that Notch ligands Dll1 and Jg1 were expressed in purified B cells themselves (33). However, other studies have failed to identify Notch ligand expression in B cells and instead detected expression in a variety of other splenic cells. One study found that Notch ligands Dll1 and Jg2 are expressed on red pulp macrophages and more weakly on CD11c^+^ dendritic cells of the mouse spleen using flow cytometry (30). Dll1 expression was also detected on erythroblasts in the same study. However, radiation bone marrow chimeras have shown that Dll1 expression on radiation-resistant stromal cells is required for formation of mouse marginal zone B cells (41). This result indicates that Notch ligand expression on hematopoietic-derived cell types such as macrophages and DC is not required at least for some Notch-dependent steps.

**Table 3:** Notch Ligand protein expression in murine cells.

	Conventional Dendritic cells	Follicular dendritic cells (FDC)	Macrophages	Endothelial cells	Erythroblasts
Dll1	+	+ *	+	+	+
Dll2	+	ND	ND	ND	ND
Dll4	+	ND	+	+	ND
Jg1	+	+ *	+	+	+
Jg2	+	ND	+	ND	ND

(+) expressed, (−) nor expressed. ND, not determined.

* Human FDC also express these Notch ligands.

Another study making use of lacZ knockin mouse strains that express the lacZ enzyme under the control of the regulatory elements of the Dll1, Dll4, or Jg1 genes showed that these genes are expressed partially overlapping patterns in endothelial cells of blood vessels and/or the marginal sinus (31). Dll1 in particular was found in blood vessels within the marginal zone of the spleen. Bone marrow chimeras confirmed that the lacZ-expressing cells were non-hematopoietic in origin (31). Stromal fibroblastic reticular cell (FRC) subsets also express Notch ligands and can contribute to Notch signaling in B cells. FRC can be subdivided into several subsets based on localization, marker gene expression and function. One type of FRC are follicular dendritic cells (FDC), which are known to be crucial in organizing B cell follicles, capturing surface-bound antigen and stimulating germinal center reactions (42). In keeping with this, immunostaining of mouse spleen detected Dll1 expression in FDC of germinal centers as well as the splenic marginal zone, while Jg1 was found only in the MZ (34). Interestingly, this study also suggests that different cells in the MZ express Jg1 versus Dll1, because co-staining with antibodies to both proteins failed to detect many co-expressing cells. The importance of FRC in presenting Notch ligands for MZ B cell development was shown by a study in which Dll1 was deleted in FRC or in CD11c+ DC or endothelial cells (43). Deletion of Dll1 in FRC, but not in DC or endothelial cells led to the complete loss of marginal zone B cells. Therefore, Notch ligand-expressing FRC are crucial for mouse MZ B cell development and may also play a role in other B cell differentiation steps in secondary lymphoid organs. But it remains possible that non-FRC cells that express Notch ligands (such as endothelial cells, macrophages or DC) could be involved in other B cell responses.

Notch ligand expression has also been studied in human lymphoid tissues. Yoon et al. demonstrated that Dll1 and Jg1 are expressed in human tonsil tissue (38). Expression of these ligands was found on follicular dendritic cells, similar to the expression of Dll1 on mouse FDCs. In the human spleen, non-lymphoid cells located in the marginal zone region have been shown to express Dll1 (44). Therefore, in both mice and humans, non-hematopoietic cells seem particularly important expressors of Notch ligands.

## Notch Signaling Represses B Lineage Commitment

**Figure 4:**
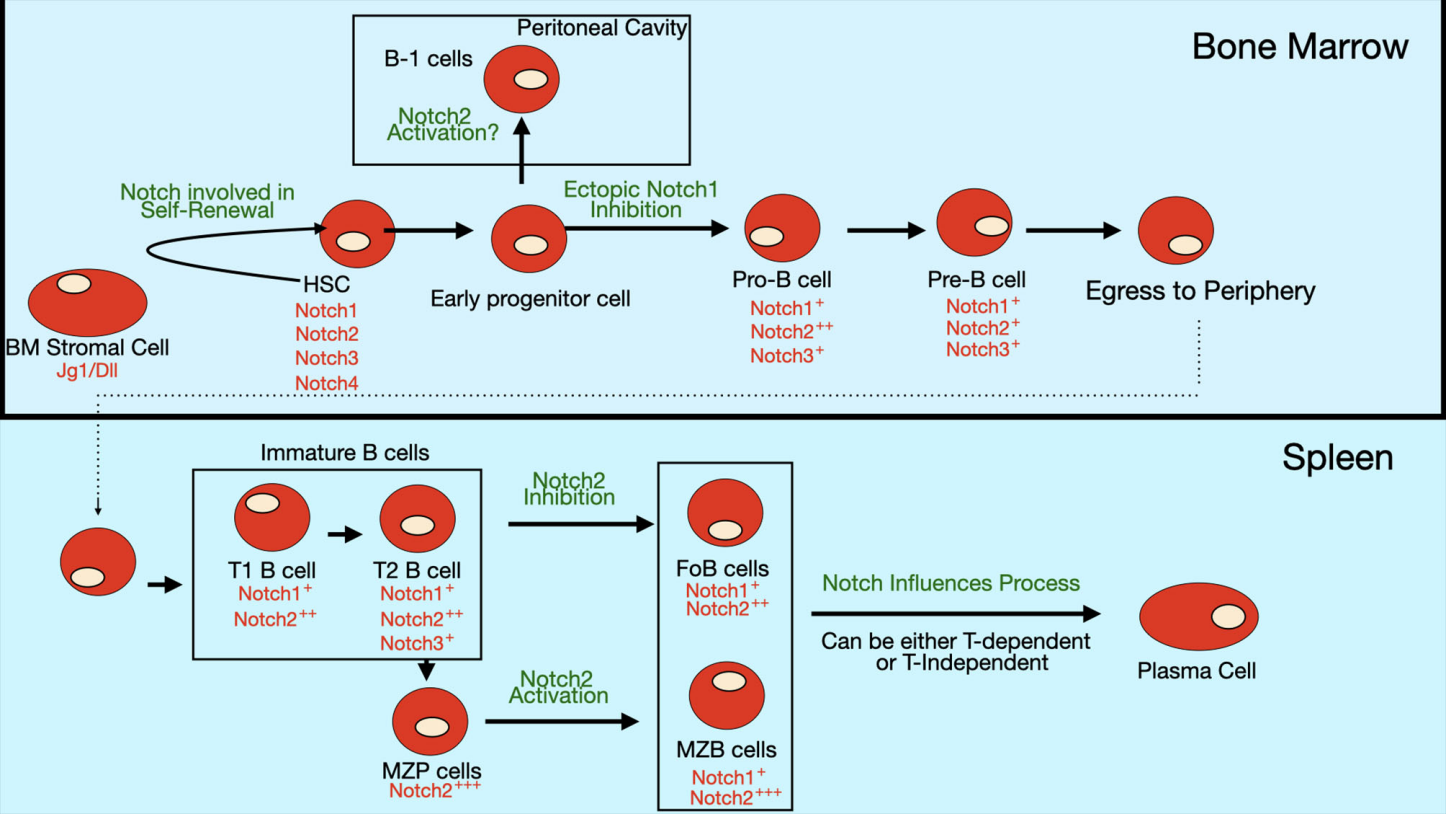
Notch expression and role in B cell differentiation. Expression of Notch receptors and ligands (red text) on bone marrow B cell progenitors and peripheral B cell subsets. Notch signaling is thought to be involved in self-renewal of hematopoietic stem cells (HSCs). The differentiation of early progenitor cells to pro-B cells is inhibited by ectopic Notch signaling, though evidence is still lacking that endogenous Notch signaling controls this step. Some evidence suggests that Notch2 activation may regulate commitment to the B-1 subset, but this pathway is still controversial since there are contradictory findings in different studies. In the periphery, Notch2 signaling is required for specification of marginal zone (MZ) B cells, while it inhibits formation of follicular (FO) B cells. Notch signaling also stimulates the differentiation of mature B cells into antibody-secreting cells (ASCs).

Hematopoietic stem cells (HSC) in the bone marrow undergo a series of differentiation steps that lead to formation of various progenitor cell populations including lymphoid-primed multi-potent progenitors (LMPP), early lymphoid progenitors (ELP) and common lymphoid progenitors (CLP). Some of these progenitor cells leave the bone marrow and travel to the thymus, where they encounter Notch ligands that trigger activation of the Notch1 receptor. Activation of Notch1 signaling in these progenitor cells is crucial for development of T cells (28, 45, 46). Ectopic Notch signaling driven by retroviral expression of the Notch1 intracellular domain in bone marrow progenitors inhibits hematopoietic progenitors from developing into B lineage committed cells in mice (47). Similarly, exposure of cultured human hematopoietic progenitors to Notch ligands Dll1 or Dll4 inhibits their differentiation to the B cell lineage, although exposure to Notch ligand Jg1 does not inhibit B cell differentiation (48, 49). Together, these results suggest that Notch signaling in the bone marrow environment has the potential to block B cell development from precursor cells (**Figure 4**). However, further study is needed to determine whether endogenous Notch signaling plays a role in this process, since the studies to date have only examined situations where Notch signaling was aberrantly activated in these progenitor populations. Constitutive expression of Notch1 intracellular domain (NICD) causes ectopic differentiation of T cells in the bone marrow (47). On the other hand, enforced expression of Deltex1, an inhibitor of Notch signaling, in hematopoietic progenitors results in B cell development at the expense of T cell development (50).

One proposed mechanism by which it does this is by promoting degradation of the transcription factor E2A, which is required for B cell differentiation (51). This process is mediated through ubiquitination and proteosomal degradation, following E2A phosphorylation by MAP kinases (52). E2A is also important in T cells. Interestingly, Notch signaling does not promote E2A degradation in T cell progenitors due to their inherent low basal levels of MAP kinase activity. In this way, Notch signals have an inhibitory effect on B cell lineage commitment, while allowing T cell fate decisions to be unaltered. Another proposed mechanism for Notch-mediated inhibition of B cell differentiation is its ability to interfere with binding of the transcription factor EBF to target genes (53). Like E2A, EBF is also required for B cell differentiation (54, 55). Notch signaling also controls expression of transcription factor LRF, another factor required for B cell differentiation (56). Transcription factor Pax5, also known as the B cell lineage specific activator protein (BSAP), is another fundamental regulator of B cell development. Pax5 expression in hematopoietic stem cells and early progenitors by knockin into the endogenous Ikaros locus promotes B-cell development at the expense of T-cell development (57). One mechanism by which it may do so is by Pax5-mediated repression of Notch1 expression. A summary of the expression pattern of Notch receptors and their roles in bone marrow B cell development are shown in **Figure 4**.

## Notch Regulation of MZ B cell Differentiation

B cells can be subdivided into two main categories, B-1 B cells and B-2 B cells. B-1 B cells have been best studied in mice, where it was shown that they are produced mainly during fetal and early postnatal life, self-renew and are localized largely in body cavities such as the peritoneal cavity (58). B-2 B cells are derived from bone marrow hematopoietic progenitors and do not self-renew being instead replenished constantly by newly-generated immature B cells from the bone marrow. B-2 cells are localized in secondary lymphoid organs, such as spleen and lymph node and can be further subdivided into marginal zone and follicular subtypes (59). Follicular B cells recirculate *via* the bloodstream and lymphatics moving between spleen and lymph nodes and can also enter other tissues in response to inflammatory stimuli. When in the spleen and lymph node, follicular B cells are localized in B cell follicles, as their name implies. Marginal zone B cells do not circulate, but rather remain in the spleen localized outside of the B cell follicle in the marginal zone area. Marginal zone B cells are specialized for responding to blood borne antigens, since they are in close contact with blood flowing through the marginal sinus of the spleen (60).

Development of marginal zone, but not follicular B cells, requires Notch signaling (**Figure 4**). The first data supporting the role of Notch signaling in MZ B cells was deletion of a floxed allele of RBJ-Jκ in B cells using CD19-Cre (61). These mice showed a dramatic reduction in MZ B cell numbers, while follicular B cells and B-1 B cells were normal. This was accompanied by reduced numbers of B cells expressing high levels of CD1d and CD9, which are markers expressed on cells committed to become MZ B cells (61). As shown in **Table 1**, Notch2 levels are highest in splenic B cells. This is consistent with the fact that Notch2 is the important Notch receptor regulating MZ B cell development, because deletion of Notch2 in B cells results in absence of MZ B cells (27). The level of Notch2 on the surface of the cell is important, because even heterozygous mice that carry one functional copy of Notch2 show a diminished number of MZ B cells (27, 62). Notch2+/- mice have also been reported to show a reduction in B-1 B cells (62), while mice with a floxed allele of Notch2 crossed to CD19-Cre mice show normal B-1 numbers (27). The reason for this difference in B-1 phenotype between the two strains remains unknown. Constitutive expression of either the Notch1 intracellular domain or the Notch2 intracellular domain is capable of driving B cell differentiation towards the marginal zone fate, although the Notch2 intracellular domain seems to do so more strongly (63, 64). Knockout of Mint, an inhibitor of Notch signaling, leads to enhanced Notch signaling and a reduction in follicular B cells and increase in marginal zone B cells (65).

Dll1 is the crucial Notch ligand for specification of the marginal zone B cell fate, as deletion of Dll1 abrogates MZ B cell formation (66). Even mice with heterozygous loss of Dll1 demonstrated reduced MZ B cells. Interestingly though, development of MZ B cells in lupus-prone NZB/W mice is less dependent on Dll1 (30). In another autoimmune model, the non-obese diabetic (NOD) mouse that develops type I diabetes, heterozygosity for Notch2 does not lead to as significant a reduction in MZ B cells as is typically seen in Notch2+/- mice on a non-autoimmune prone C57BL/6 background (67). Together these studies imply that the autoimmune milieu may provide signals that can overcome the need for the Notch2/Dll1 pathway in MZ B cell development.

Humans have marginal zone B cells as well, though they exhibit some differences as compared to their mouse counterparts (68). Unlike mouse MZ B cells, human MZ B cells frequently have mutated variable (V) regions in their antibody genes. They also express CD27, suggesting that they are antigen-experienced (68). Human MZ B cells can arise also in patients with mutations in CD40 or CD40 ligand indicating that they are at least in part activated in a T cell-independent fashion (69). Human MZ B cells may participate in responses to blood-borne bacteria, similar to mouse MZ B cells (68). Human transitional B cells isolated from cord blood and stimulated for 4 days with TLR9 ligand (CpG ODN) differentiated into cells with characteristics of MZ B cells and expressed high levels of Notch2 (70). A precursor population for human MZ B cells has been identified and shown to be responsive to DLL1 (44). Patients with Alagille syndrome involving mutations in Notch2 have a variety of developmental defects and show reduced MZ B cells (44).

Several other components of the Notch signaling pathways have also been shown to regulate generation of MZ B cells in mice. Loss of Nicastrin (a subunit of *γ*-secretase) (71), Adam10 (12) or Maml1 (72, 73), block MZ B cell generation. Loss of nicastrin also impairs B-1 cell development. Combined loss of Lfng or Mfng or of all three Fringe family members reduces MZ B cell numbers (31, 74). Deletion of Notch pathway inhibitor MINT results in increased MZ B cell numbers (75). On the other hand, deletion of Hes1 does not affect MZ B cell formation, though it impacts Notch-dependent T cell development (76).

The kinase Taok3, which is required for normal expression of Adam10 on transitional B cells in the spleen, is necessary for MZ B cell development (77). Interestingly, Taok3-dependent Adam10 upregulation on transitional B cells marks their commitment to become marginal zone B cells. Another signaling pathway controlling Adam10 levels on transitional B cells is the Gαi pathway (78). Deletion of Gαi2 or both Gαi2 and Gαi3 (encoded by the genes Gnai2 and Gnai3) in B cells leads to loss of marginal zone B cells. This is accompanied by poor expression of Adam10 on the surface of transitional B cells. Gαi signaling is triggered downstream of G protein-coupled receptors including chemokine receptors such as CXCR4 and CXCR5. S1PR1 is another G-protein-coupled receptor that could potentially influence Gαi activation and Notch signaling in marginal zone precursors. S1PR1 is required for marginal zone B cell precursor migration into the marginal zone region of the spleen (79).

Notch signaling can regulate transcription factor activity in B cells. For instance, B cells constitutively-expressing the Notch1 intracellular domain have elevated expression of Id2, which inhibits the function of E proteins such as E2A (64). Furthermore, as described above, Notch signaling in hematopoietic progenitors results in degradation of E2A downstream triggered by MAP kinase phosphorylation (51, 52). Expressing a form of E2A that is resistant to MAP kinase phosphorylation and degradation reverses the pro-MZ differentiation effect of the constitutively-active Notch1 intracellular domain (64). These results imply that Notch-mediated suppression of E protein function is one mechanism by which it promotes development of MZ B cells. Notch signaling also induces expression of the transcription factor Fos (80). Notch2 deletion in B cells results in decreased Fos expression, while over-expression of the Notch2 intracellular domain induces Fos. In a cell culture model system, retroviral-driven expression of Fos in Notch2-deficient B cells or bone marrow was suggested to partially restore MZ development based on an increase in the percentage of CD23^lo^CD21^hi^ cells observed (80). However, it is not clear if Fos expression is a key determinant of Notch activity as B cells lacking Fos were not studied. Irf4 seems to function as a negative regulator of the Notch pathway, since Irf4-deficient B cells have elevated expression and activation of Notch2 and elevated numbers of MZ B cells (81, 82). Irf4 also suppresses induction of activation of Notch1 in BCR and CD40 stimulated B cells, as measured by Western blot for the Notch1 intracellular domain (82). Importantly, that study also shows that retention of wild-type B cells in the marginal zone is dependent on continued signaling *via* Notch2, because inhibition of Notch2 signaling results in loss of MZ B cells to the peripheral blood.

CD19 can also influence Notch pathway activity in B cells. Loss of CD19 impairs generation of MZ B cells (63, 83). This is associated with a decreased expression of cell surface Notch2 on CD19-deficient B cells and restoring Notch2 activation using a lentiviral vector results promotes MZ development of CD19-deficient cells (83). Adam family member Adam28 is expressed at high levels of the precursors on marginal zone B cells (83, 84). Loss of CD19 was associated with reduced Adam28 levels on these precursor cells. Lentiviral driven expression of Adam28 promotes Notch2 cleavage and differentiation of MZ precursors to MZ B cells, even in the absence of CD19 (83). These data implicate Adam28 in Notch2 cleavage, but as described above, other data implicate Adam10 as being the relevant Adam family member that cleaves Notch2 (12, 77). Further study is needed to clarify the roles of these two Adam proteins.

Marginal zone B cells often express BCRs that are polyreactive or autoreactive (85). Mice carrying a transgene encoding a BCR that recognizes the self-antigen keratin as well as foreign antigens present on *Candida albicans* (the TgVH3B4 transgene) have an increase in MZ B cells as compared to non-transgenic littermates (86). When these mice were crossed to mice with a B cell-specific deletion of RBP-Jκ, the resulting TgVH3B4 RBP-Jκ deficient progeny had MZ B cells, while non-transgenic RBP-Jκ mice virtually lacked MZ B cells (86). Therefore, self-reactive B cells may be able to overcome the need for Notch signaling in the differentiation pathway to MZ B cells. As described above, data obtained with lupus-prone NZB/W mice and diabetes-prone non-obese diabetic (NOD) mice also suggest that autoimmunity can partially overcome the need for Notch signaling in the development of MZ B cells (30, 67).

## Notch Interactions Causing Differentiation of B Cells to Antibody-Secreting Cells (ASCs)

Antibody-secreting cells can be formed from B cells during either T-dependent or T-independent immune responses. T-independent responses tend to generate short-lived antibody-secreting cells that remain proliferative (plasmablasts), while T-dependent germinal center reactions tend to produce longer-lived, non-proliferative and antibody-secreting cells (fully differentiated plasma cells). Long-lived plasma cells contribute in a significant way to immunological memory, since they can persist in bone marrow for decades and continue to secrete high affinity isotype-switched antibodies (87). Some data indicates a role for the Notch pathway in ASC differentiation. Several studies have examined the effects of co-culturing B cells with stromal cells expressing the Notch ligand Dll1. Santos et al. showed that CD23+ B cells (follicular B cells) activated with LPS in the presence of stromal cells expressing Dll1 gave rise to an increased number ASCs and higher titers of antibodies without an alteration in B cell proliferation (34). Similar results were obtained with anti-CD40 stimulated B cells, except that in this case Dll1 stimulated both B cell proliferation and B cell differentiation to ASCs (34, 72). Dll1 also stimulates the proliferation of BCR-stimulated B cells and BCR and CD40 co-stimulated B cells (72). These roles of Notch signaling on ASC generation are summarized in **Figure 4**. Dll1 increased isotype switching and changed the pattern of secreted antibody isotypes in stimulated B cell cultures. B cells stimulated only with anti-CD40 secreted IgM and IgG1, while B cells stimulated with anti-CD40 in the presence of Dll1 also secreted IgG2b (72). B cells stimulated with both anti-IgM and anti-CD40 proliferate, but don’t secrete much antibody. However, anti-IgM and anti-CD40 in the presence of Dll1 resulted in significant secretion of IgG1 and IgG2b. B cells stimulated with anti-CD40 + IL-4 typically secrete a large amount of IgG1 as well as some IgM and IgE. Co-culture with Dll1 expressing cells resulted in production of IgG2b along with IgG1, IgM and IgE (72). The effects of Dll1 on antibody secretion were dependent on activity of the Notch co-activator Maml1, since a dominant-negative version of this protein blocked Dll1 effects in upregulating IgG1 secretion.

Unlike Dll1, another Notch ligand Jagged1 (Jg1) was not able to induce increased ASC differentiation (34), suggesting specificity in the Notch ligand required. The effect of the Notch pathway was at a late stage of B cell differentiation after the upregulation of the plasma cell marker CD138 and could be blocked by a dominant-negative form of the Maml cofactor of the Notch signaling pathway (34, 72). Deletion of Notch1 reduces B cell antibody secretion in response to LPS stimulation (33). In this latter study, no stromal cells expressing Dll1 were used and hence Notch ligands must have been expressed by the B cells themselves or small numbers of other spleen cells that contaminated the B cell cultures (33). Interestingly, deletion of Notch1 had no effect on B cell antibody secretion in response to LPS if the B cells were not purified, but rather cultured as part of a mixture of whole spleen cells (33). This implies that an unknown factor produced by non-B cells in the cultures can substitute for the effects of Notch on ASC generation.

In another study, B cells over-expressing the Notch1 intracellular domain (and thereby mimicking constitutive Notch signaling) were reported to generate more CD138+ cells when stimulated *in vitro* with anti-IgM and anti-CD40 antibodies (32). Stimulating with anti-IgM and anti-CD40 in the presence of Dll1-expressing stromal cells resulted in an increase in the amount of IgM, IgG2a, IgG2b, and IgG3 antibodies secreted and this effect is abrogated in the absence of Notch1 (32). Using a mouse multiple myeloma model involving over-expression of the Xbp1 transcription factor, Kellner et al. discovered a post germinal center but pre-plasma cell population of mouse B cells that has high levels of Notch1 expression (88). These cells could reconstitute antibody production in B cell-deficient mice more efficiently than a terminally-differentiated ASC cell population. Together the studies above suggest that follicular B cells can upregulate Notch1 upon activation and that expression of Notch1 can stimulate generation of ASCs, either in response to T-independent stimuli (LPS) or T-dependent stimuli (anti-CD40).

Despite the studies that indicate a role for Notch signaling in ASC generation, loss of RBP-Jκ in B cells did not result in any change in ASC numbers in *ex vivo* isolated spleen, lymph node, bone marrow or Peyer’s Patch cells (61). This result suggests that Notch1 signaling in mature B cells may proceed through a non-canonical RBP-Jκ independent pathway. It is also possible that differences in purification or stimulation conditions between these various experiments may have influenced the role of the Notch pathway on ASC generation.

## Notch Effects on T Cell-Independent Immune Responses

Marginal zone and B1 cells are the main B cell types that respond to T cell-independent stimuli (89). Mice with alterations in Notch2 signaling have strong reductions in MZ B cells, suggesting they may have reduced T cell-independent responses. However, RBP-Jκ deficient mice, which lack MZ B cells, show no antibody-secretion defect in response to NP-Ficoll and even show 2-3 fold increased antigen-specific responses in response to NP-LPS (61). B-1 B cells probably account for the responses to T-independent antigens when MZ B cells are absent allowing normal T-independent responses.

Deletion of Notch1 does not impair MZ B cell development (28). However, as noted above, Notch1 deficient B cells have reduced differentiation to ASCs in response to *in vitro* stimulation with the T cell-independent stimulus LPS. This observation suggests that there might be reduced responses to T-cell independent antigens *in vivo* in mice lacking Notch1. Indeed, Notch1 deficient mice showed reduced antigen-specific IgM and IgG production when immunized with the T cell-independent type I antigen (NP-LPS) (32). There was also a reduction in antigen-specific IgG when Notch1 deficient mice were immunized with the T cell-independent type II antigen (NP-Ficoll), although this reduction was not as striking as the reduction in response to NP-LPS (32). Therefore, *in vivo* Notch1 seems more important than Notch2 for regulation of T-independent immune responses.

## Notch Effects on T Cell Dependent Immune Responses and Germinal Centers

**Figure 5:**
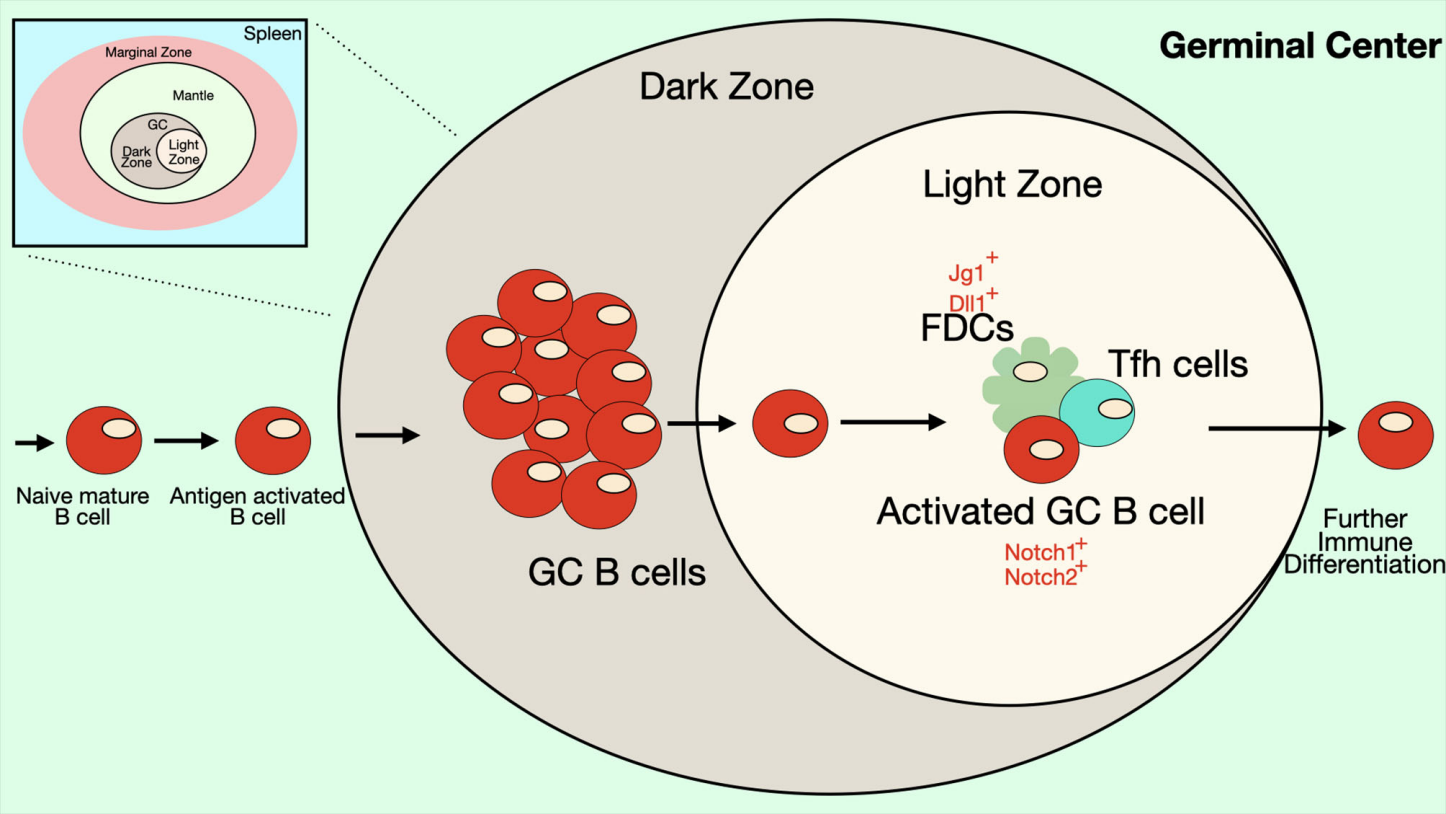
Notch signaling in the Germinal Center. Some B cell follicles in the peripheral lymphoid organs contain germinal centers (GC), where B cells rapidly proliferate and undergo somatic hypermutation, affinity maturation and isotype-switching. The germinal center has two anatomical compartments. In the dark zone of the GC, B cell centroblasts rapidly proliferate while also undergoing somatic hypermutation. In the light zone of the GC, B cell centrocytes compete for binding antigen on follicular dendritic cells (FDCs) and receive signals from T follicular helper (Tfh) cells that promote survival and differentiation. Together these processes select for B cells with BCRs that recognize antigen with high affinity. FDCs express DSL ligands that may play a role in signaling during germinal center reactions. In mice, GC B cells express both Notch1 and Notch2 receptors, though in humans expression of these Notch receptors is low on GC B cells. Notch signaling in GC B cells has been shown to antagonize the effects of BCL6 and may play a role in differentiation of GC B cells into ASCs. Differentiation of Tfh cells also requires Notch signaling.

Follicular B cells are the main B cell type that responds to T cell-dependent stimuli, although marginal zone B cells can also participate in these reactions. Unlike MZ B cells, specification of the follicular B cell lineage is not controlled by Notch signaling. However, Notch signaling can influence T-dependent germinal center (GC) responses in which follicular B cells play the most prominent role (**Figure 5**). As described above, stimuli that mimic signals found in GCs (anti-CD40 or anti-IgM and anti-CD40 antibodies) can stimulate B cell differentiation to ASCs (32, 34, 72). Notch ligands are expressed by FDCs and Notch1 and Notch2 receptors are expressed by GC B cells (34, 38). GC B cells cultured with an FDC cell line (HK cells) along with CD40 ligand, IL-2 and either IL-4 or IL-21 undergo proliferation, differentiation to plasmablasts and secretion of antibodies and the addition of an inhibitor of the *γ*-secretase (DAPT) inhibits these processes (38). As was seen with T cell-independent responses, loss of RBP-Jκ does not interfere with antigen-specific antibody production in response to the T cell-dependent antigen NP-CGG (61). This may indicate that Notch effects in GC B cells are *via* a non-canonical Notch pathway. On the other hand, immunizing mice that have B cell-specific expression of dominant-negative Maml1 with the T cell-dependent antigen NP-CGG resulted in production of fewer NP-specific plasma cells compared to controls (72). Dominant-negative Maml1 mice also had decreased frequencies and numbers of IgG1+ B220-low plasma cells (72).

Notch signaling in GC B cells may be influenced by the transcription factor BCL6, which is crucial for GC B cell formation. In *Xenopus laevis*, the Notch1 intracellular domain was shown to interact with BCL6 (90), displacing Maml proteins from the Notch intracellular domain. Because Maml proteins are co-activators of Notch-dependent transcription, their displacement results in weaker Notch-mediated transcriptional events. BCL6 also functions to recruit the co-repressor BCoR to the NICD, thus further suppressing Notch-dependent transcription (90). Another mechanism by which BCL6 could inhibit Notch signaling in GC B cells was described by Valls et al. in human follicular lymphomas, a tumor derived from GC B cells (40). In these cells, the BCL6 protein binds to regulatory elements of the Notch2 gene and other Notch pathway genes (Maml1, Maml2, RBP-Jκ and Hes1) to directly repress their expression. BCL6 and Notch pathway genes show an inverse correlation in expression patterns in primary human and murine GC B cells (40). BCL6-dependent repression of Notch2 seems to be crucial for GC maintenance, since tamoxifen-inducible expression of the Notch2 intracellular domain abrogates GC formation in mice (40). On the other hand, BCL6-targeting compounds or gene silencing leads to the induction of Notch2 activity. Together, these data indicate two possible mechanisms by which BCL6 may interfere with Notch signaling, either by binding to the NICD and displacing the co-activator Maml, while recruiting the co-repressor BCoR, or by directly binding to and repressing Notch pathway genes. Both mechanisms may take place in GC B cells. The ability of BCL6 to interfere with Notch signaling may be needed to prevent premature differentiation of GC B cells to ASCs, a process induced by Notch activity.

Notch signaling can also have B cell-extrinsic effects that influence GC formation. T follicular helper cells (Tfh) are a subset of CD4+ T cells that are specialized for helping B cells in germinal center reactions. Like GC B cells, Tfh cells express the transcription factor BCL6 and this transcription factor is crucial for the ability of Tfh cells to differentiate properly and provide B cell help (91). Combined deletion of both Notch1 and Notch2 using CD4-Cre results in impaired Tfh cell generation, while total CD4 cells, Tregs, memory CD4 T cells and naïve CD4 T cells are all normal (92). The decrease in Tfh cells results in impaired GC formation in response to immunization with T-dependent antigens or parasite infection. There was also impaired generation of high-affinity antigen-specific IgG antibodies. The few Tfh cells that develop in Notch1/Notch2-deficient mice express low levels of Tfh markers such as BCL6, IL-21, c-Maf and CXCR5 (92). Therefore, Notch signaling can have both B cell-intrinsic and B-cell extrinsic effects in the GC.

Germinal center-derived B cell lymphomas often have excessive and dysregulated Notch signaling caused by activating mutations (93). Many mutations in Notch receptors are localized in the PEST domain and result in premature truncation thereby blocking PEST-mediated degradation of NICD and causing prolonged Notch signaling. To test potential roles for this dysregulated Notch signaling, Arima et al. developed a mouse model in which the intracellular domain of Notch1 (NICD) was expressed in B cells under the control of the germinal center specific AICDA-Cre. This resulted in an increased percentage of GC cells, while other B cell subsets were normal (94). In this mouse model, B cells produced the cytokine IL-33, which led to secondary effects in the T cell compartment including an expansion of Treg and Th2 cell subsets and a decrease in cytokine production by Th1 and CD8+ T cells (94). Thus, increased Notch signaling in B cells can lead to B cell-extrinsic effects on the immune response.

## Conclusions

In this review, we’ve summarized published studies implicating the evolutionarily-conserved Notch signaling pathway in regulating B cell differentiation and functional responses. These studies clearly show an important influence of the Notch pathway in guiding appropriate differentiation at many steps of B cell development from the hematopoietic progenitor cells in the bone marrow to terminally-differentiated B cells in immune responses. There are various compounds that can be used to target both the canonical and noncanonical pathways of Notch signaling and could potentially influence immune responses. However, the involvement of Notch signaling in crucial developmental decisions in a huge variety of cell types in the body makes it very difficult to specifically target B cell responses, unless methods can be developed to deliver Notch activators or inhibitors directly to B cells without affecting other nearby cell types. A number of interesting questions remain to define concerning Notch pathway activity in B cells. Notch2 is clearly required for the MZ fate specification, but Notch1 seems more important in ASC differentiation. However, Notch2 has not been inducibly-deleted in mature B cells to allow studies to test its function in generation of ASCs from already committed MZ and follicular B cells. Another interesting question is whether non-canonical Notch signaling plays an important role in B cell differentiation. Notch2 seems to trigger the canonical pathway to specify the MZ B cell fate, since loss of RBP-Jk interferes with this process. However, in T dependent and T-independent immune responses, B cells lacking Notch1 show a defect, while those lacking RBP-Jκ do not. This implies that a non-canonical pathway may be more important at this stage. Future studies will be important to address some of these interesting conundrums.

## Author Contributions

MG collected information, wrote the manuscript and prepared the figures. LG-S edited and approved the final version. All authors contributed to the article and approved the submitted version.

## Funding

This work was supported by grants from the Lupus Research Alliance and the National Institute of Allergy and Infectious Disease (NIAID R01 AI122720).

## Conflict of Interest

The authors declare that the research was conducted in the absence of any commercial or financial relationships that could be construed as a potential conflict of interest.
